# Postoperative radiotherapy for prostate cancer: Morbidity of local-only or local-plus-pelvic radiotherapy

**DOI:** 10.1007/s00066-017-1215-9

**Published:** 2017-09-19

**Authors:** Cora Waldstein, Wolfgang Dörr, Richard Pötter, Joachim Widder, Gregor Goldner

**Affiliations:** 1 Department of Radiation Oncology, Comprehensive Cancer Center, General Hospital of Vienna, Medical University of Vienna, Währinger Gürtel 18–20, 1090 Vienna, Austria; 2 Christian-Doppler Laboratory for Medical Radiation Research for Radiooncology, Medical University of Vienna, Vienna, Austria

**Keywords:** Survival analysis, Genitourinary system, Toxicity, Gastrointestinal tract, Actuarial incidence rate, Überlebensanalyse, Urogenitalsystem, Toxizität, Gastrointestinaltrakt, Aktuarische Inzidenzrate

## Abstract

**Purpose:**

The aim of this work was to characterise actuarial incidence and prevalence of early and late side effects of local versus pelvic three-dimensional conformal postoperative radiotherapy for prostate cancer.

**Materials and methods:**

Based on a risk-adapted protocol, 575 patients received either local (*n* = 447) or local-plus-pelvic (*n* = 128) radiotherapy. Gastrointestinal (GI) and genitourinary (GU) side effects (≥grade 2 RTOG/EORTC criteria) were prospectively assessed. Maximum morbidity, actuarial incidence rate, and prevalence rates were compared between the two groups.

**Results:**

For local radiotherapy, median follow-up was 68 months, and the mean dose was 66.7 Gy. In pelvic radiotherapy, the median follow-up was 49 months, and the mean local and pelvic doses were 66.9 and 48.3 Gy respectively. Early GI side effects ≥ G2 were detected in 26% and 42% of patients respectively (*p* < 0.001). Late GI adverse events were detected in 14% in both groups (*p* = 0.77). The 5‑year actuarial incidence rates were 14% and 14%, while the prevalence rates were 2% and 0% respectively. Early GU ≥ G2 side effects were detected in 15% and 16% (*p* = 0.96), while late GU morbidity was detected in 18% and 24% (*p* = 0.001). The 5‑year actuarial incidence rates were 16% and 35% (*p* = 0.001), while the respective prevalence rates were 6% and 8%.

**Conclusions:**

Despite the low prevalence of side effects, postoperative pelvic radiotherapy results in significant increases in the actuarial incidence of early GI and late GU morbidity using a conventional 4‑field box radiotherapy technique. Advanced treatment techniques like intensity-modulated radiotherapy (IMRT) or volumetric modulated arc radiotherapy (VMAT) should therefore be considered in pelvic radiotherapy to potentially reduce these side effects.

## Zusammenfassung

**Ziel:**

Ziel der vorgestellten Arbeit ist es, die Häufigkeit früher und später Nebenwirkungen nach postoperativer Bestrahlung von Prostatakarzinompatienten zu analysieren. Verglichen wurden dabei die Nebenwirkungen von lokaler Bestrahlung mit denen nach lokaler Bestrahlung plus Beckenbestrahlung (4-Felder-Box-Technik).

**Material und Methoden:**

Basierend auf einem risikoadaptierten Protokoll erhielten 575 Patienten nach Prostatektomie entweder eine konventionelle lokale Bestrahlung (*n* = 447) oder eine Beckenbestrahlung (*n* = 128). Gastrointestinale und urogenitale Nebenwirkungen ≥ Grad 2 wurden prospektiv anhand der RTOG/EORTC-Klassifikation erhoben. Verglichen wurden die maximale Morbidität sowie die aktuarischen Inzidenz- und Prävalenzraten in beiden Gruppen.

**Ergebnisse:**

Bei lokaler Bestrahlung waren die mediane Nachsorgezeit 68 Monate und die mittlere Dosis 66,7 Gy, bei Beckenbestrahlung waren die mediane Beobachtungszeit 49 Monate und die lokale Dosis bzw. die Beckendosis 66,9 und 48,3 Gy. Frühe gastrointestinale Nebenwirkungen wurden bei 26 % (lokal) bzw. bei 42 % (Becken) beobachtet (*p* < 0,001), eine späte gastrointestinale Morbidität bei jeweils 14 % in beiden Gruppen (*p* = 0,77). Die aktuarischen Fünfjahresinzidenzraten waren 14 bzw. 14 %, die Prävalenz lag bei 2 bzw. 0 %. Frühe urogenitale Nebenwirkungen wurden bei 15 bzw. 16 % beobachtet (*p* = 0,96), eine späte urogenitale Morbidität bei 18 bzw. 24 % (*p* = 0,001). Die aktuarischen Fünfjahresinzidenzraten betrugen 16 bzw. 35 %, während die Prävalenz bei 6 bzw. 8 % lag.

**Schlussfolgerung:**

Trotz der generell niedrigen Prävalenz strahlenbedingter Nebenwirkungen führte die konventionelle 4‑Felder-Box-Beckenbestrahlung zu einem signifikanten Anstieg der aktuarischen Inzidenz früher gastrointestinaler und später urogenitaler Nebenwirkungen in der postoperativen Behandlung von Patienten mit Prostatakarzinom. Bei der Bestrahlung von Beckenfeldern sollten daher moderne Techniken wie VMAT („volumetric modulated arc radiotherapy“) oder IMRT („intensity-modulated radiotherapy“) zum Einsatz kommen, sodass diese Nebenwirkungen potenziell verringert werden.

Postoperative radiotherapy for prostate cancer (PCa) is currently regarded as standard of care in patients with an increased risk for recurrence after radical prostatectomy (RP). Typical risk factors are infiltration of the seminal vesicles, extraprostatic extension, positive surgical margins, a high Gleason Score or a high pre-RP prostate-specific antigen (PSA) level [1, 2].

Three randomised studies (SWOG 8794, EORTC 22911, ARO 96-96/AUOAP 09/95) demonstrated improved biochemical recurrence-free survival with local adjuvant radiotherapy compared with surgery alone [3–7]. Moreover, long-term follow-up of the SWOG S8794 trial showed improved overall survival for patients with pT3N0M0 PCa [4]. However, about 25% of the patients will suffer biochemical failure even after receiving adjuvant local radiotherapy. Therefore, extending the postoperative treatment volume to include pelvic lymph nodes has been suggested based on the assumption that patients with an increased risk of lymph node involvement could benefit from elimination of microscopic disease [8, 9].

However, the role of elective radiotherapy of the pelvic nodal regions in clinically node-negative patients is controversial [10–12], since side effects may be more frequent and clinical benefit has not yet been established definitively. As the median life-expectancy after treatment for PCa is 13.8 years [13], it is particularly important to carefully assess long-term toxicity after pelvic radiation.

After radiotherapy for prostate cancer, urinary and bowel urgency and/or incontinence, as well as dysuria and rectal bleeding are the most frequently reported side effects [14–19]. Due to the increased irradiated volume in pelvic lymph node radiotherapy, an increased incidence and/or severity and/or an extended duration of adverse events may be expected. For primary external beam radiation therapy, RTOG 9413 compared the toxicity between prostate only and whole pelvic radiotherapy using three-dimensional (3D) conformal techniques, and a nonsignificant increase in the rate of early and late toxicity was observed after whole pelvic radiotherapy (WPRT) [20, 21]. However, toxicity data in the postoperative setting comparing pelvic and local-only radiotherapy are scarce.

The aim of the present study was to investigate the difference in terms of incidence and prevalence rates as well as the duration of late side effects between local and pelvic postoperative external beam radiation therapy in the treatment of PCa. We used monocentric prospectively assessed data of a consecutive cohort of postoperative patients undergoing local or pelvic radiotherapy based on an institutional risk-adapted protocol and being followed-up for up to 15 years. Side effects were prospectively evaluated at regular time intervals according to European Organization for Research and Treatment of Cancer/Radiation Therapy Oncology Group (EORTC/RTOG) criteria in a standardised fashion.

## Patients and methods

### Patients

In this prospective cohort study, 961 patients received postoperative radiotherapy between 1994 and 2011. In general, patients underwent radical prostatectomy combined with standard lymphadenectomy, which is limited to the obturator fossa and/or external iliac lymph nodes and typically comprised removal of 3–4 lymph nodes. Androgen deprivation therapy was given to high-risk patients, if one or more of the following factors was present: prostate-specific antigen (PSA) score > 20 ng/ml at baseline, Gleason score of 8–10, or clinical stage T2C or higher.

According to our institutional policy, the Roach formula estimating the probability of lymph node involvement in the primary setting was employed to make the indication for WPRT using baseline data prior to surgery (cutoff, lymph node involvement probability ≥ 15%) [22]. Patients with an unknown Gleason Score but with a high grading (G3) or a high PSA value prior to radiotherapy (≥20 ng/ml) also received WPRT. Patients who had a low risk of lymph node involvement (<15%) or who were 80 years or older or who had a history of inflammatory bowel disease, a history of colorectal surgery, or a Karnofsky performance score < 80% received local radiotherapy only. Before entry, all patients underwent bone scintigraphy, CT or MRI scans of the abdomen and the pelvis and PSA testing. Participants with pathologically positive lymph nodes (*n* = 59), distant metastases (*n* = 19), alternative radiotherapy techniques (*n* = 17) or with a follow-up of less than 18 months (*n* = 274) were excluded. In addition, patients with local radiation doses <65 Gy (*n* = 14), those with pelvic radiation doses <45 Gy (*n* = 2), or >50.4 Gy (*n* = 1) were excluded. Thus, 447 pathologically node-negative patients receiving local radiotherapy and 128 patients receiving local-plus-pelvic lymph node radiotherapy were included in the current analysis.

### Radiotherapy

All patients were treated using a 3-dimensional four-field box technique with individualised collimation. The target volume was delineated according to ICRU-report 62 [23]. For patients undergoing local radiotherapy, the clinical target volume (CTV) included the prostate bed. For patients undergoing pelvic nodal radiotherapy, the CTV included the prostate bed and the iliac internal, external and communis lymph nodes up to the aortic bifurcation. The safety margin around the CTV for both groups was 10–12 mm in all directions. Both groups received a dose of 65–74 Gy in 1.8–2 Gy fractions to the prostate bed, and the patients with pelvic radiotherapy received an additional 45–50.4 Gy in 25–28 fractions to the pelvic lymph nodes.

### Morbidity assessment

Patients were seen weekly or every two weeks during radiotherapy, every 3–6 months for the first year after radiotherapy and at least once per year thereafter. Gastrointestinal (GI) and genitourinary (GU) side effects were prospectively scored by a study physician according to EORTC/RTOG criteria [24]. Early side effects were assessed until 3 months after the end of radiotherapy. GI symptoms included stool frequency, stool consistency, faecal incontinence, rectal pain, cramping, rectal mucous discharge, urgency of defecation and rectal bleeding. GU side effects included frequency, urgency, incontinence, dysuria and haematuria [24]. The durations of late GI and GU side effects of grade ≥ 2 were calculated from first diagnosis to their last occurrence.

### Statistical analysis

To estimate the risk of developing a defined maximum grade of side effects at least once at any time-point during follow-up, reporting actuarial incidence rates is a common practice [25]. However, actuarial incidence rates alone do not provide any information about the duration or the possibility of recovery of the assessed side effects. Prevalence rates indicating the relative percentage of patients suffering from late side effects at a defined time-point, may provide clinically more meaningful information [26–30].

As the competing risk (death) was negligibly low (<8% over a period of more than 10 years), actuarial incidence rates of GI and GU side effects of grade ≥ 2 were calculated using the Kaplan–Meier method, and differences between curves were estimated using the log-rank statistic. Maximum morbidities were tabulated and compared using the chi square (χ^2^) test. In addition, the prevalence of side effects of grade ≥ 2 was calculated for each follow-up time-point and compared using Fisher’s exact test. The duration of side effects was statistically analysed using unpaired t‑tests. All time parameters were calculated from the last day of radiotherapy. Calculations were performed using Prism Version 4 (GraphPad Software, Inc., San Diego, CA, USA). The significance level was set at *p* < 0.05.

## Results

A total of 575 patients were included in this analysis. For patients undergoing local radiotherapy (*n* = 447), the median follow-up was 68 (range 18–203) months, and the mean dose was 66.7 (66–74) Gy. Median follow-up of patients undergoing pelvic nodal radiotherapy (*n* = 128) was 49 (19–159) months, with a mean local and pelvic dose of 66.9 (65–74) Gy and 48.3 (45–50.4) Gy respectively. Patient characteristics are summarised in Table 1.

**Table 1:** Pretreatment and treatment-related patient characteristics

		Local RT *n* = 447 (%)	Pelvic RT *n* = 128 (%)
*Age (years)*	Mean	65	66
	Range	45–82	44–80
*pT stage*	pT 2	183 (41%)	48 (38%)
	pT 3	230 (51%)	73 (57%)
	pT 4	27 (6%)	4 (3%)
	T X	7 (2%)	3 (2%)
*PSA (ng/ml)*			
*Prior to surgery*	Average (min–max)	13 (0.21–290)	22 (1.2–1000)
*Post surgery*	Average (min–max)	0.23 (0–7.18)	0.26 (0–4.3)
*Prior to RT*	Average (min–max)	1.39 (0–111.4)	1.55 (0–24.34)
*Gleason score*	2–6	154 (34%)	16 (12.5%)
*(Based on surgery)*	7	137 (31%)	45 (35%)
	8–10	78 (17.5%)	52 (40.5%)
	Unknown	78 (17.5%)	15 (12%)
*Roach formula %*	Mean	16	25
	Range	0–66	0–100
*Additional HT*	Yes	174 (39%)	56 (44%)
	No	271 (60%)	71 (55%)
	Orchiectomy	1 (0.5%)	0 (0%)
	Unknown	1 (0.5%)	1 (1%)

*RT* radiotherapy, *PSA* prostate specific antigen, *HT* hormone therapy

Early GI side effects ≥grade 2 were detected in 26% (115/447) and in 42% (54/128) of patients receiving local or pelvic radiotherapy respectively (*p* = 0.0004). Early GU side effects ≥ grade 2 (maximal rates) were detected in 15% (69/447) and 16% (20/128) of patients with local or pelvic radiotherapy respectively (*p* = 0.96). The early GI and GU sideeffects rates, by grade, are given in Table 2.

**Table 2:** Early gastrointestinal (*GI*) and urogenital (*GU*) adverse events separated in grades using the European Organization for Research and Treatment of Cancer (EORTC)/Radiation Therapy Oncology Group (RTOG) classification

	Local RT-GI *n*/%	Pelvic RT-GI *n*/%	Local RT-GU *n*/%	Pelvic RT-GU *n*/%
*Grade 0*	153 34%	22 17%	199 44%	41 32%
*Grade 1*	179 40%	52 41%	179 40%	67 52%
*Grade 2*	113 25%	53 41%	66 15%	20 16%
*Grade 3*	2 1%	1 1%	3 1%	0 0%
*Grade 4*	0 0%	0 0%	0 0%	0 0%

*RT* radiotherapy, *GI* gastrointestinal, *GU* genitourinary, *n* number of patients

**Fig. 1:**
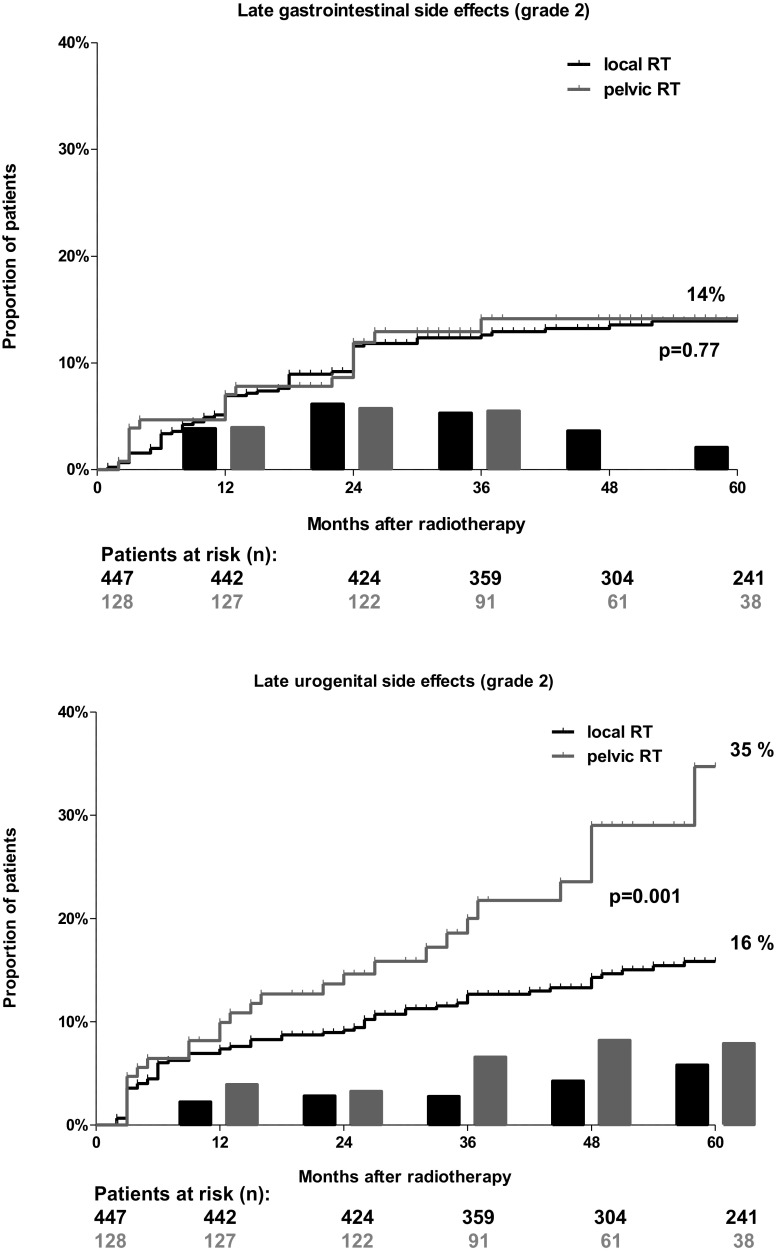
Combination of actuarial incidence and prevalence rate of late gastrointestinal (Gl) and genitourinary (UG) side effects ≥ grade 2 (European Organization for Research and Treatment of Cancer/Radiation Therapy Oncology Group classification) after prostate cancer radiotherapy (RT). The curve represents the actuarial incidence rate and the bars represent the prevalence of side effects at each follow-up visit. The constant increase in the incidence rate and the rather stable prevalence rates indicate that a constantly increasing number of patients is suffering from—however—mainly temporary side effects

Late GI adverse events ≥ grade 2 were detected in 14% (63/447) of patients receiving local, and in 14% (18/128) of patients receiving pelvic radiotherapy (*p* = 0.77). The corresponding 5‑year actuarial incidence rates were 14% for both radiotherapy techniques; in contrast, the prevalence rates at 5 years were 2% and 0% (Fig. 1) respectively (n. s.). Regarding severe (grade 3) toxicity, there were five events for local and two events for pelvic radiotherapy. All patients presented with persistent rectal bleeding and needed argon plasma coagulation of rectal wall telangiectasiae.

Late GU morbidity ≥ grade 2 was detected in 18% (80/447) of patients receiving local radiotherapy, compared to 24% (31/128) for pelvic radiotherapy (*p* = 0.001). The corresponding 5‑year actuarial incidence rates were 16% and 35% respectively (*p* = 0.001), while the prevalence rates after 5 years were 6% and 8% respectively (Fig. 1; *p* = 0.6). The late GI and GU side effects rates are displayed in Table 3, for each individual grade. Regarding severe (grade 3) toxicity, there were sixteen events for local and five events for pelvic radiotherapy. In the majority of events (90%) urethral stricture following urethral extension occurred. Two patients required coagulation of severe telangiectasia. One patient developed a contracted bladder with low capacity following an ileal conduit urinary diversion (grade 4 GU toxicity).

**Table 3:** Late gastrointestinal gastrointestinal (GI) and urogenital (GU) adverse events separated in grades using the Radiation Therapy Oncology Group/European Organization for Research and Treatment of Cancer classification

	Local RT-GI *n*/%	Pelvic RT-GI *n*/%	Local RT-GU *n*/%	Pelvic RT-GU *n*/%
*Grade 0*	292 65%	70 55%	258 58%	72 56%
*Grade 1*	92 21%	40 31%	109 24%	25 20%
*Grade 2*	58 13%	16 12%	64 14%	25 20%
*Grade 3*	5 1%	2 2%	16 4%	5 4%
*Grade 4*	0 0%	0 0%	0 0%	1 1%

*RT* radiotherapy, *GI* gastrointestinal, *GU* genitourinary, *n* number of patients

The mean duration (± standard deviation) of late GI side effects ≥ grade 2 was 16.7 ± 24.7 months after local radiotherapy and 8.8 ± 9.6 months after pelvic radiotherapy (*p* = 0.10). The mean duration (± standard deviation) of late GU side effects ≥ grade 2 was 16.0 ± 24.1 months after local radiotherapy and 11.9 ± 17.9 months after pelvic radiotherapy (*p* = 0.39).

## Discussion

In this study we investigated the incidence, prevalence, and duration of early and late side effects in postoperative patients with PCa after local versus pelvic radiotherapy. Our main finding was a significant difference for the incidence of early GI side effects (≥grade 2) in patients receiving pelvic radiotherapy compared to patients receiving local-only radiotherapy (42% versus 26%, *p* = 0.0004). The incidence of late GI side effects (≥grade 2) was, however, not significantly different between both groups (14% versus 14%, *p* = 0.77). Concerning GU side effects, no statistically significant difference for the incidence of early ≥ grade 2 side effects was detected between both groups (15% versus 16%, *p* = 0.96). In contrast, the incidence of late GU morbidity ≥ grade 2 was significantly different with 18% in patients receiving local radiotherapy compared to 24% for pelvic radiotherapy (*p* = 0.001).

In general, the published data on early and late side effects of WPRT are inconclusive which is in line with the clinical controversy regarding this particular kind of therapy. In particular, data on postoperative radiotherapy are scarce. Two prior studies evaluated the incidence and prevalence of side effects in the setting of primary radiotherapy for PCa. In the prospective randomised trial RTOG 94-13 (70.2 Gy to the prostate and 50.4 Gy to the whole pelvis), a trend for higher rates of early and late grade 3 GI complications in the WPRT combined with neoadjuvant and concurrent hormonal therapy arm was observed, without, however, reaching statistical significance, with *p* = 0.06 and *p* = 0.09 respectively [21]. In addition, in the updated 2007 RTOG analysis with a mean follow-up duration of 6.6 years, a higher risk for early and late morbidity was observed with a significantly higher incidence (5% versus 1%, *p* = 0.002) of late grade 3 or higher GI reactions in the WPRT arm [31]. With the inclusion of lower-grade morbidity (grade 2 or higher), our study exhibited increased sensitivity for the detection of early GI and GU morbidity; this may have led to higher rates of side effects in our cohort. An analysis by Aizer et al. [32], who administered 75.6 Gy to the prostate (4-field box/intensity-modulated radiotherapy, IMRT) and 45 Gy to the whole pelvis (4-field box), retrospectively investigated a cohort of 227 patients comparing WPRT and prostate-only radiotherapy (PORT). In agreement with our study, patients undergoing WPRT had increased early GI toxicity (*p* = 0.048), but no significant difference in early GU toxicity (*p* = 0.09). No difference in late morbidities was observed, but the follow-up duration of that study (34 months for PORT and 25 months for WPRT patients) was more limited. However, as, postoperative RT patients are expected to have higher intestinal toxicities as such [33], it is difficult to compare both series with our study.

To the best of our knowledge, only a few studies compared WPRT and PORT toxicity in the postoperative setting. A retrospective analysis by Deville et al. [34] compared 36 patients treated with WPRT versus 31 patients treated with PORT using IMRT technique (70.2 Gy to prostate bed, 45 Gy to pelvic lymph nodes) and showed a significant increase in early GI side effects (*p* = 0.001), but no difference in acute GU and late GI/GU side effects. This small study had a limited median follow-up of 25 (12–44) months. Van Praet et al. [35] prospectively investigated 48 node-positive patients treated with WPRT, combined with androgen deprivation and 239 node-negative patients treated with PORT (70.2 Gy to prostate bed, 45 Gy to pelvic lymph nodes). WPRT significantly increased early and late GI/GU side effects. Their study had a median follow-up of 24 (12–60) months for WPRT and 48 (12–56) months for PORT patients, which may have been a limitation in evaluating late toxicity.

Our study was specifically designed to evaluate GI and GU morbidity according to predefined, standardised criteria. At each follow-up visit, patients were interviewed by a study physician according to EORTC/RTOG criteria. This may have resulted in a more accurate and robust assessment of radiation side effects in a patient population that is generally doing well clinically and may not report GI or GU disturbances on their own initiative.

Clinical manifestations of late urogenital side effects, as shown in our study, could be reduced by using advanced treatment techniques like IMRT or volumetric modulated arc radiotherapy (VMAT). Although it is necessary to include sensitive structures such as the prostatic urethra, trigonum vesicae and the bladder neck into the target volume, recent data suggest a reduction of urogenital morbidity with IMRT [36, 37]. Respecting normal tissue tolerances, IMRT and VMAT could more accurately spare organs at risk, especially the upper bladder, intestine, bowel and the rectal wall.

It is interesting to observe that long-term prevalence rates of radiation side effects (60 months after radiotherapy and onwards) remained at relatively low levels. This low prevalence rate in combination with a steadily rising actuarial incidence rate indicate that the majority of patients recovered from treatment-associated morbidity. Unlike other studies, we also investigated the duration of the complications. In relation to the typically long life-expectancy of PCa patients, the duration of side effects was generally short, and—moreover—not significantly associated with WPRT. In 2012, Schmid et al. [30] also demonstrated long-term recovery from GI and GU side effects after local-only radiotherapy.

### Limitations

One of the limitations of our study is the disparity in terms of disease stage and follow-up periods of the two treatment groups, which is due to increasing referrals of patients with higher-risk disease in more recent periods. However, especially actuarial incidence rates are minimally affected by this difference in follow-up durations, especially as even in the WPRT group the median follow-up was 48 months. But still, some degree of sampling bias in the analysis of the long-term morbidity cannot be excluded with certainty. Moreover, treatment allocation (local versus pelvic radiotherapy) was not randomised; however, predefined risk-dependent criteria were used to allocate patients to the respective treatment modalities. Due to the naturally uneven distribution of disease characteristics in the patient population, the sample sizes of the two groups were not equal. A further limitation of this study is that systematic data on patient comorbidities are not available.

## Conclusion

Our study demonstrates a statistically significant increase in early GI, and late GU, toxicities in patients undergoing adjuvant pelvic nodal radiotherapy for prostate cancer. Advanced radiotherapy techniques may be instrumental in reducing or avoiding long-term morbidity in an active population with long-term survival perspectives.
